# Epidemiology and Microbiologic Characterization of Nosocomial Candidemia from a Brazilian National Surveillance Program

**DOI:** 10.1371/journal.pone.0146909

**Published:** 2016-01-25

**Authors:** André Mario Doi, Antonio Carlos Campos Pignatari, Michael B. Edmond, Alexandre Rodrigues Marra, Luis Fernando Aranha Camargo, Ricardo Andreotti Siqueira, Vivian Pereira da Mota, Arnaldo Lopes Colombo

**Affiliations:** 1 Department of Medicine, Division of Infectious Diseases, Universidade Federal de São Paulo, São Paulo, SP, Brazil; 2 Division of Medical Practice, Hospital Israelita Albert Einstein, São Paulo, SP, Brazil; 3 Instituto Israelita de Ensino e Pesquisa Albert Einstein, Hospital Israelita Albert Einstein, São Paulo, SP, Brazil; 4 Department of Internal Medicine, University of Iowa Carver College of Medicine, Iowa City, IA, United States of America; 5 Laboratório Central, Hospital São Paulo, São Paulo, SP, Brazil; V.P.Chest Institute, INDIA

## Abstract

Candidemia is a growing problem in hospitals all over the world. Despite advances in the medical support of critically ill patients, candidiasis leads to prolonged hospitalization, and has a crude mortality rate around 50%. We conducted a multicenter surveillance study in 16 hospitals distributed across five regions of Brazil to assess the incidence, species distribution, antifungal susceptibility, and risk factors for bloodstream infections due to *Candida* species. From June 2007 to March 2010, we studied a total of 2,563 nosocomial bloodstream infection (nBSI) episodes. *Candida* spp. was the 7^th^ most prevalent agent. Most of the patients were male, with a median age of 56 years. A total of 64 patients (46.7%) were in the ICU when candidemia occurred. Malignancies were the most common underlying condition (32%). The crude mortality rate of candidemia during the hospital admission was 72.2%. Non-*albicans* species of *Candida* accounted for 65.7% of the 137 yeast isolates. *C*. *albicans* (34.3%), *Candida parapsilosis* (24.1%), *Candida tropicalis* (15.3%) and *Candida glabrata* (10.2%) were the most prevalent species. Only 47 out of 137 *Candida* isolates were sent to the reference laboratory for antifungal susceptibility testing. All *C*. *albicans*, *C*. *tropicalis* and *C*. *parapsilosis* isolates were susceptible to the 5 antifungal drugs tested. Among 11 *C*. *glabrata* isolates, 36% were resistant to fluconazole, and 64% SDD. All of them were susceptible to anidulafungin and amphotericin B. We observed that *C*. *glabrata* is emerging as a major player among non-*albicans Candida* spp. and fluconazole resistance was primarily confined to *C*. *glabrata* and *C*. *krusei* strains. *Candida* resistance to echinocandins and amphotericin B remains rare in Brazil.

Mortality rates remain increasingly higher than that observed in the Northern Hemisphere countries, emphasizing the need for improving local practices of clinical management of candidemia, including early diagnosis, source control and precise antifungal therapy.

## Introduction

Candidemia is a growing problem in tertiary care hospitals all over the world. It is a cause of late sepsis and risk factors include broad-spectrum antibiotic therapy, ICU stay for more than 72 hours, immunosuppressive therapy, parenteral nutrition, and multiple invasive medical procedures [1,2,3,4]. A recent study conducted in 183 US medical centers showed that *Candida* ranked as the most common cause of bloodstream infections [5].

Despite advances in the medical support of critically ill patients, hematogenous candidiasis is still considered difficult to diagnose, leads to prolonged hospitalization, has a mortality rate of around 50% and is a financial burden to health care systems [2,6,7].

The incidence of candidemia may vary substantially from region to region, but numbers appears to be stable or increasing in most series [7–11]. Although *C*. *albicans* is still considered the major pathogen associated with candidemia, infections due to non-*albicans* species are increasing and may present resistance to some antifungal agents [1,12–18].

Recent studies have documented increasing rates of fluconazole and echinocandin resistance in the US and European countries [18–20]. Indeed, in addition to *C*. *glabrata* and *C*. *krusei*, fluconazole resistance has also been documented among *C*. *tropicalis* and *C*. *parapsilosis* strains in different regions [15,20–22].

The epidemiology of candidemia has been extensively studied in the United States and Europe, but not in Latin America, and there is a lack of data on echinocandin resistance in Latin America [5,6,13]. Recently, Bizerra et al [23] published the first case of echinocandin resistance in Brazil illustrating the relevance of conducting continuous surveillance activities to identify changes in incidence rates, susceptible populations, etiologic agents, and antifungal resistance in our region.

We conducted a multicenter surveillance study in 16 hospitals geographically distributed across five regions of Brazil to assess the incidence, species distribution, frequency of antifungal resistance, and risk factors for bloodstream infections due to *Candida* species.

## Materials and Methods

Brazilian Surveillance and Control of Pathogens of Epidemiologic Importance (BrSCOPE), involved 16 participating public and private hospitals throughout the five regions in Brazil (North, Northeast, Middle-East, Southeast and South). The study was approved by the Institutional Review Board (IRB) at each participating site and it was submitted to the final approval of the IRB from Universidade Federal de Sao Paulo. The requirements for informed consent was waived by the IRB of Universidade Federal de Sao Paulo in accordance with the Code of Federal Regulations and Privacy Rule.

The participating centers in this study were: Hospital Israelita Albert Einstein, Universidade Federal de Sao Paulo (UNIFESP), Hospital 9 de Julho, Santa Casa de Porto Alegre, Hospital Conceição, Hospital de Base de Brasiília, Hospital Walter Cantidio, Hospital de Diadema, Instituto de Oncologia Pediatrica-IOP/GRAAC, Santa Casa do Pará, Hospital Espanhol, de Salvador, Hospital do Coração de Natal, Hospital da UNIMED de Natal, Hospital das Clinicas de Goiânia, Hospital do Rim e Hipertensão and Universidade Federal do Triangulo Mineiro.

### Study design

The Brazilian SCOPE (BrSCOPE) was a surveillance project based at Universidade Federal de São Paulo Brazil that included 16 hospitals geographically distributed across five regions of Brazil. Clinical data and microbiological features of the first episode of nosocomial bloodstream infection from 12 June 2007 to 31 March 2010 were collected and analyzed.

A nosocomial BSI was diagnosed if 1 or more cultures of blood sampled at least 48 h after admission yielded a pathogenic organism. If the bloodstream isolate was a potential skin contaminant (e.g., diphtheroid, *Propionibacterium* spp., *Bacillus* spp., coagulase-negative staphylococci, or micrococci), the presence of an intravascular catheter and the initiation of targeted antimicrobial therapy were required for the diagnosis, as well as at least 1 of the following findings: temperature of >38.0°C or <36°C, chills, and/or systolic blood pressure of <90 mm Hg. BSI episodes that represented relapses were excluded [11]. In this study, we are only analyzing the infections due to *Candida* spp.

Clinical data were concurrently collected by local infection control practitioners using a standardized case report form and forwarded to the coordinating center, along with all eligible episodes of BSI caused by yeasts. The data that were routinely collected included the patient’s gender, location at the onset of BSI (ICU versus non-ICU ward), clinical service at the onset of BSI, and predisposing clinical conditions, as well as the identifications and antimicrobial susceptibilities of the causative pathogens and status at discharge. Predisposing clinical conditions that were routinely recorded included neutropenia (defined as an absolute neutrophil count of <1,000/mL), peritoneal dialysis or hemodialysis, and presence of intravascular catheters (i.e., central lines, arterial catheters, or peripheral intravenous catheters).

### Microbiological methods

Blood cultures were processed at the participating hospitals using automated systems. The identification of blood isolates was performed by routine methods in use at the affiliated laboratories as API-ID32 (Biomerieux, Marcy l’Étoile France) or automated systems (Vitek 2 Biomerieux). All affiliated laboratories were Brazilian Society of Clinical Pathologists certified, and all microbiological methods used were consistent with current Clinical and Laboratory Standards Institute (CLSI) recommendations. The yeast isolates were sent to the reference laboratory, the Special Laboratory of Mycology at Universidade Federal de São Paulo, for confirmation of species identification and antifungal susceptibility testing.

All identifications were confirmed by MALDI-TOF using the Microflex LT (Bruker Daltonics, Bremen Germany) and the software Biotyper 3.0. according to the manufacturer’s recommendations. Antifungal susceptibility testing was performed using the broth microdilution method according to CLSI document M27-A3, using current CLSI MIC interpretative criteria (CLSI, M27-S4) [24,25]. *Candida parapsilosis* ATCC 22019 and *Candida krusei* ATCC 6258 were used as quality controls.

The antimicrobial agents tested were all provided by the manufacturers as pure powder for analysis: fluconazole (Pfizer Incorporated, New York, NY, USA), voriconazole (Pfizer Incorporated, New York, NY, USA), anidulafungin (Pfizer Incorporated, New York, NY, USA) and amphotericin B (Sigma-Aldrich, St Louis, MO, USA).

Statistical analysis. Distribution Analysis of continuous variables with median and mean determinations was performed. Differences in proportions were compared using a chi- square test or Fisher’s exact test, as appropriate. All tests of significance were 2 tailed; p-value was set at 0.05. All statistical analyses were done using SPSS software (SPSS version 21).

## Results

From June 2007 to March 2010, we studied a total of 2,563 nosocomial bloodstream infection (nBSI) episodes with 2,688 microorganisms reported by the 16 participant centers. *Candida* spp. was the 7^th^ most prevalent agent, accounting for 137 monomicrobial BSIs and one polymicrobial BSI.

Characteristics of the 137 patients with nBSIs caused by *Candida* spp. at the 16 Brazilian hospitals are demonstrated in Table 1. Most of the patients were male, with a median age of 56 years. Most of patients needed intensive care support (64.2%) and 46.7% were in the ICU when candidemia occurred.

**Table 1:** Demographics and clinical characteristics of the 137 patients with *Candida* spp. monomicrobial nosocomial bloodstream infections.

Parameters	No
**Demographics**	
Male	71 (51.8%)
Age (median)	56 y.o
**Hospitalization**	
Time to candidemia*	29 days
ICU admission**	88 (64.2%)
ICU at the time of candidemia	64 (46.7%)
**Underlying Conditions**	
Malignancy	44 (32.1%)
Gastrointestinal	26 (18.9%)
Neurologic	11 (8.0%)
Respiratory	9 (6.5%)
Renal	9 (6.5%)
Hepatic	8 (5.8%)
Cardiovascular	7 (5.1%)
Trauma	6 (4.3%)
Transplantation (solid organ)	5 (2.9%)
Transplantation (bone marrow)	2 (1.4%)

*Time to candidemia: time from hospital admission to first culture positive for *Candida* spp.

** ICU—Intensive Care Unit.

Concerning predisposing factors, 68 patients (49.6%) were mechanically ventilated, 8 were receiving hemodialysis and 121 (88.3%) had central venous catheters at the time of the diagnosis of candidemia.

The mean time between admission and first nBSI caused by *Candida* spp. was 29 days.

Malignancies were the most common underlying condition, but neutropenia was rare (<1%).

The crude mortality of candidemia patients during the hospital admission was 72% (53% for non-ICU and 85% for ICU patients (p<0,01), Table 2.

**Table 2:** Crude mortality of patients with candidemia stratified by venue.

	Total (137)	ICU *(64)	Non ICU (73)	Private Hospital **(28)	Non-private Hospital (109)
**Crude Mortality**	72.2% (99)	85.0% (55)	53.0% (39)	75.0% (21)	66.9% (73)

*ICU vs. non-ICU—p-value <0.01 (OR = 5.3 95%CI 2.2–13.6).

**Private hospital vs. non-private hospital—p-value = 0.42 (OR = 1.5 95%CI 0.5–4.3).

Of the 137 *Candida* isolates causing monomicrobial nBSI, non-*albicans* species accounted for 65.7%. The rank order of the major *Candida* spp. isolated was *C*. *albicans* (34.3%), *Candida parapsilosis* (24.1%), *Candida tropicalis* (15.3%), *Candida glabrata* (10.2%), *Candida krusei* (1.5%), *Candida pelliculosa* (1.5%), *Candida lusitaniae* (0.7%), *Candida famata* (0.7%), and *Candida guilliermondii* (0.7%).

Only 47 out of 137 *Candida* isolates were sent to the reference laboratory (LEMI) for further confirmation of species and antifungal susceptibility testing.

The identification at species level was correct for all isolates tested by the sentinel centers and 6 strains initially identified as *Candida* spp. were determined to be *C*. *tropicalis* (4), *C*. *glabrata* (1) and *C*. *albicans* (1). Consequently, *C*. *tropicalis* accounted for 18% of all 137 candidemia episodes.

The 36 *C*. *albicans*, *C*. *tropicalis* and *C*. *parapsilosis* isolates were susceptible to the 4 antifungal drugs tested.

Among 11 *C*. *glabrata* isolates, 4 isolates were resistant to fluconazole, and 7 fluconazole susceptible dose-dependent (SDD). All were susceptible to anidulafungin and amphotericin-B (see Table 3).

**Table 3:** *In vitro* susceptibility of 47 *Candida* spp. strains against 4 antifungal agents.

Species/MIC (μg/mL)	Drug	0,03	0,06	0,125	0,25	0,5	1,0	2,0	4,0	8,0	16	32	64	>64
***C*. *albicans* (N = 14)**	Amphotericin B					6	8							
	Fluconazole			13	1									
	Voriconazole	12		2										
	Anidulafungin	14												
***C*. *tropicalis* (N = 16)**	Amphotericin B						16							
	Fluconazole			2		2	7	5						
	Voriconazole	13	2	1										
	Anidulafungin	16												
***C*. *glabrata* (N = 11)**	Amphotericin B						11							
	Fluconazole							1*	1*		3*	2*	1**	3**
	Voriconazole			1	6	4								
	Anidulafungin	11												
***C*. *parapsilosis* (N = 6)**	Amphotericin B					5	1							
	Fluconazole				1	1	3	1						
	Voriconazole	4	2											
	Anidulafungin					4	2							

Only 47 out of 137 *Candida* spp. were available for antifungal susceptibility testing.

* SDD—Susceptible Dose Dependent (CLSI M27 S4)).

** R—Resistant (CLSI M27 S4).

## Discussion

Few multicenter studies have been published in Brazil addressing the incidence of candidemia, susceptible populations, crude mortality rates, etiology and rates of *in vitro* antifungal resistance [10,11,26,27].

In our series, *Candida* spp. was the 7^th^ most prevalent cause (5.6%) of nosocomial bloodstream infection among all pathogens studied in SCOPE Brazil where 137 monomicrobial *Candida* nBSI were characterized [26].

It is important to emphasize that the incidence of candidemia is probably underestimated in the present study considering that: 1) we excluded putative patients who could develop candidemia after bacteremia since we have evaluated only the first episode of bloodstream infection reported after hospital admission and 2) automated blood culture is far more sensitive for the detection of bacteremia than fungemia.

It is also important to highlight that we included in this study a total of 16 tertiary care hospitals serving high complexity patients with good clinical practices for treating septic patients, including the collection of blood cultures for all patients with suspicion of sepsis. In addition, according to the SCOPE study methodology, all investigators were asked to select for the study only the first episode of nosocomial BSI. Therefore, subsequent episodes of BSIs were not considered. Consequently, it is expected that a substantial number of patients that could further develop candidemia after bacteremia were censored. This could explain the low number of blood cultures recorded per year.

A similar epidemiologic study, SCOPE USA, revealed that *Candida* spp. were the 4^th^ most prevalent agent (4% of the total) [28]. Data recently reported by a national point prevalence survey conducted in 183 centers in the US revealed that *Candida* has become the most common microorganism causing BSI. This finding is probably related to the substantial reduction of primary bacteremia due to implementation of bundles to reduce BSI [5].

Another multicenter study showed that *C*. *glabrata* is now emerging as a relevant pathogen in Brazil, accounting for 10% of all episodes. [12] Corroborating this finding, *C*. *glabrata* accounted for 10% of all candidemia episodes in the present series. Otherwise, *C*. *tropicalis* and *C*. *parapsilosis* strains remain the non-*albicans Candida* species more prevalent among candidemia episodes. [6,12,15]

Of note, differently from series in the US and Europe, *C*. *tropicalis* was highly prevalent also among non-cancer patients. This finding had already been noted in previous series published in our region [2,8,11].

The reasons for the emergence of non-albicans species are not completely understood, but some risk factors may include the presence of central vascular catheters, total parenteral nutrition, cancer, neutropenia and antifungal selective pressure [4,11,29].

The crude mortality of candidemia in our study was 72.2%, much higher than that found in Scope USA 1995–2002 (39.2%) [28], Wisplinghoff et al 2014 (38.1%) [30] and other Brazilian series [8,10,11].

As reported in previous Brazilian series [10,11,13], cancer was the most common underlying condition followed by gastrointestinal and neurologic diseases. Vascular catheters were the most important risk factors in our patients]. ICU admissions at the onset of candidemia was 46.7%, a rate that is also similar to other series. In this scenario, discrepancies of mortality documented in the present series in contrast to previous publications may be partially explained by differences in the median ages of the populations evaluated. The median age of the patients in our study was 56 years, higher than that reported by Nucci et al and Colombo et al, (41 and 36 years old, respectively [2,13].

Indeed, according to other published series, mortality rates of candidemia in Brazil are very high, usually exceeding 50% [2,11]. Several factors may explain this phenomenon including: severity of underlying conditions (32.1% had malignancy as the principal underlying condition), high APACHE score at the time of diagnosis (47% patients requiring ICU care at the onset of candidemia), late diagnosis of fungemia, and suboptimal management of candidemia by the clinical staff.

In addition, we cannot exclude the possibility that the diagnosis was made at a late stage of disease when patients are less likely to respond to antifungal therapy. Another hypothesis to be considered is the suboptimal treatment of sepsis by *Candida* either by the overuse of fluconazole in critically ill patients instead of echinocandins, as well inadequate source control of the fungal infection. [31]

Considering our hypothesis that higher mortality rates could be partially related to the aging of the susceptible population, in the present series only 16% of the patients were under 12 years old, a finding that is completely differently from other multicenter series published in which 32% (Colombo et al) and 40% (Nucci et al) of candidemic patients were children [8,11].

Regarding the rate of antifungal resistance, we were able to test only 47 of 137 isolates. Despite this limitation, our data is consistent with previous Brazilian series where fluconazole resistance is basically confined to *C*. *glabrata* and *C*. *krusei* strains. Finally, Candida resistance to echinocandins and amphotericin B remains rare in Brazil and Latin America [11–13].

In summary, we observed that *Candida* spp. is one of the most important pathogens causing nosocomial BSI where *C*. *glabrata* is emerging as a major player among non-albicans *Candida* spp, a reality different than we observed a decade earlier.

Mortality rates remain higher than that observed in the Northern Hemisphere countries, emphasizing the importance of early diagnosis, appropriate control of the infectious source, and appropriate antifungal therapy.
